# Job Satisfaction Among Employees After a Merger: A Cross-Sectional Survey in the Local Health Unit of Sardinia Region, Italy

**DOI:** 10.3389/fpubh.2021.798084

**Published:** 2021-12-09

**Authors:** Claudia Isonne, Angelo Nardi, Pasquale de Soccio, Alessandro Zerbetto, Monica Giffi, Alessandro Sindoni, Daniela Marotta, Valentina Baccolini, Giuseppe Migliara, Rosario Mete, Carolina Marzuillo, Paolo Villari, Giovanni Salis, Fulvio Moirano, Corrado De Vito

**Affiliations:** ^1^Department of Public Health and Infectious Diseases, Sapienza University of Rome, Rome, Italy; ^2^Complex Structure Director for Training, Research and Organizational Change, ATS Sardinia, Nuoro, Italy; ^3^General Manager, ATS Sardinia, Sassari, Italy

**Keywords:** job satisfaction, merger & acquisition, employees, local health units, occupational well-being, survey

## Abstract

Job satisfaction plays an important role in healthcare organization and management; it is critical for maintaining and improving staff efficiency and consequently the quality of care provided. Organizational restructuring processes, including mergers, are likely to affect job satisfaction levels, but evidence of the impact they have is surprisingly scarce. The aim of the study was to describe a methodology used to measure job satisfaction of the employees at a Local Health Unit (LHU) in Italy immediately after a merger and to assess the determinants associated with any reduction in worker satisfaction. The study was conducted among employees of the LHU of the Sardinia Region in July 2018, after a merger of eight subregional LHUs had taken place. The entire staff was enrolled, of which a total of 1,737 employees were surveyed. We used a questionnaire exploring socio-demographic and working characteristics of the employees, the various areas related to job satisfaction and interviewee opinions on the merger process. Multivariable stepwise backward logistic regression models were built to identify factors independently associated with lower job satisfaction. The results of a multivariable analysis showed that lower job satisfaction was more likely in employees with an administrative role (aOR: 2.34, 95% CI: 1.37–4.00) or a career demotion (aOR: 1.84, 95%CI: 1.11–3.03). High levels of mental stress were strongly associated with lower job satisfaction (aOR: 5.64, 95%CI: 4.16–7.64). “More equity of employee rewards” was the only example of a set of responder suggestions found to be associated with lower job satisfaction (aOR: 2.30, 95%CI: 1.51–3.47). Generally, responders showed a good level of job satisfaction—and this was also the case following the merger—but some job profile determinants were strongly associated with low employee satisfaction. The results of the study highlighted several challenging areas and critical issues relating to working conditions. Further surveys are required to confirm these results and to monitor their evolution over time.

## Introduction

Job satisfaction represents an important issue for healthcare management and organization, as it is well known to be associated with both patient and staff outcomes. Thus, healthcare worker satisfaction is positively associated with patient satisfaction and healthcare quality, such as patient adherence to treatment protocols and hospital-adjusted mortality ratios (1–3). On the other hand, job dissatisfaction seems to have an impact on job-related outcomes, such as burnout, turnover and absenteeism (4–6). Issues relating to working conditions (7, 8), including psychological factors, wages, job security, relationships with colleagues and supervisors, and career opportunities (9) have been identified as potential determinants of job satisfaction. Therefore, any event leading to a change in working conditions, like an organizational restructuring, could influence job satisfaction (10, 11).

Among such events, the merger of healthcare structures is highly likely to modify the organizational climate and thus job satisfaction (12). Although in recent decades many countries have undertaken mergers of healthcare institutions, little is known about the impact of such mergers on job satisfaction, either in the hospital setting or in local health units (LHUs) (13, 14). Moreover, most quantitative studies have focused only on the impact on costs and efficiency (15), such that data on staff perception are limited (16). However, the small amount of evidence available from qualitative research suggests that mergers can lead to a loss of trust in the psychological contract (i.e., the implicit obligations and expectations between employers and employees) because of poor communication (17), delays in service development, and difficulties in learning and sharing “good practice” (12). Furthermore, various unfavorable consequences and potential disadvantages have been described as resulting from a merger, including lower morale, stress due to the fear of job loss and clashes of corporate culture (18).

Recently in Italy, there has been a policy of merging LHUs to obtain economies of scale and thereby to reduce pressure on healthcare budgets (19). However, to our knowledge there have been no studies of the effect of this LHU unification process on the job satisfaction of workers. In this paper, we focused on the case of the Italian LHU “Azienda Tutela della Salute” (ATS) of the Sardinia Region, which arose from the merger of eight subregional LHUs (ASSL Cagliari, ASSL Carbonia, ASSL Lanusei, ASSL Nuoro, ASSL Olbia, ASSL Oristano, ASSL Sanluri, ASSL Sassari) and covers a population of 1,653,135 residents. The purpose of the study was to describe the methodology used to assess the job satisfaction of LHU employees immediately after the merger. We then explore and assess socio-demographic and job-related variables associated with lower worker satisfaction in the context of the new institutional arrangements.

## Methods

### Data Collection

We studied the “Azienda per la Tutela della Salute” (ATS), the Local Health Unit (LHU) of the Sardinia Region, which was established in accordance with Italian Regional Law 17/2016 and began operating on 1 January 2017 after the merger of eight previously existing LHUs. The entire staff of the ATS (16,000 employees) was invited to take part in the study through the administration of an online questionnaire in July 2018, 18 months after the merger came into effect.

Researchers generated alphanumeric codes that were matched to individual pay slips by ATS personnel. To access the survey, the respondents used their alphanumeric code and an anonymous link, which were provided in their pay slip together with a cover letter giving details of the project. All precautions regarding privacy protection were taken in accordance with Italian law and all data were processed with due regard to the principles of transparency, proportionality, impartiality and legality. In order to present and explain the questionnaire to LHU Sardinia workers and to make them aware of the aims and purposes of the analysis, on-site training meetings were organized by the researchers in conjunction with LHU management. These meetings, which took place at the headquarters of LHU Sardinia, also aimed to instruct the largest number of “facilitator members” on the objectives and methods of the survey. The trained facilitators were, in turn, asked to inform their colleagues about the opportunity to reply to the questionnaire.

### Questionnaire

The questionnaire was adapted to fit the purposes of this study from a version previously developed by Gigantesco et al. (20). It consisted of 58 questions divided into three sections. The first section (11 items) investigated the socio-demographic and working characteristics of the employees in order to assess their job profile. The second section comprised 42 items in six areas relating to working conditions and a further question on overall employee job satisfaction. The items were grouped as follows: (i) work organization (13 items), (ii) work schedule (two items), (iii) relationships (five items), (iv) workplace (three items), (v) physical stress (four items), and (vi) mental stress (15 items). For each question, the answer was structured as a five-point Likert-type scale, ranging from one (worst condition) to five (best condition). To quantify the overall job satisfaction of the employee, an item providing an answer on a 10-point analog quantitative scale (1: the worst working conditions; 6: acceptable working conditions; 10: the best working conditions) was adopted. The third section consisted of three items that assessed interviewee opinion on the merger process, together with a final open-ended question. Specifically, these questions investigated changes in professional profile and a judgement of the quality of the healthcare services provided. Lastly, at the end of the questionnaire, the interviewee was invited to make suggestions that might help improve working conditions.

### Statistical Analysis

Descriptive statistics for socio-demographic variables, working condition items and employee suggestions were calculated. For each group of questions, the overall score was calculated and dichotomized according to the 75th or 25th percentile to identify the more- and less-satisfied groups of employees: in particular, the 75th percentile was used to identify the more-satisfied employees in the areas of work organization, relationships, environmental conditions and work schedule in order to assess how positive perception in these areas protects against dissatisfaction; the 25th percentile was used to identify the less-satisfied employees in the areas of mental and physical stress, in order to assess how stress contributes to dissatisfaction. For the item on overall job satisfaction (scored on a 10-point analog scale), the 25th percentile was used as the cut-off to identify employees with lower job satisfaction.

Univariate analysis was performed to assess possible associations between each variable and having a lower job satisfaction. Multivariable stepwise backward logistic regression models were built to identify factors independently associated with lower job satisfaction (Model 1) and to assess employee suggestions independently associated with it (Model 2). Variables were included in the models when the *p*-value derived from the univariate analysis was lower than 0.25 or when they were considered relevant to the outcome. Adjusted odds ratio (aOR) and 95% confidence intervals (CIs) were calculated. All statistical analyses were performed with STATA 15 (StataCorp LLC, 4905 Lakeway Drive, College Station, Texas, USA). A *p*-value <0.05 was considered statistically significant.

## Results

The socio-demographic characteristics of the 1,737 respondents are summarized in Table 1 (response rate: 10.8%). The mean age was 51.4 years (standard deviation: 9.2 years), with the most represented age category being 51–60 years (42.4%). Over 60% of the workers were women, and more than half of the entire sample had a university degree (56.5%), whereas only 9.0% had an elementary-middle school educational level. As for job type, the participants were mainly healthcare workers (77.4%), followed by administrative staff (13%), and showed a similar distribution between the hospital (50.9%) and the health districts (49.1%). Almost 29% of the respondents had worked for LHUs for <5 years and the vast majority had a permanent employment contract (92.2%) with no change in professional profile due to the merger (91.3%). The largest category of workers were formerly at the Cagliari LHU (26.3%) followed by those from Sassari LHU (17.6%). There were 303 (17.4%) employees who had a management role.

**Table 1:** Socio-demographic and occupational characteristics of the respondents (*N* = 1,737).

**Variables**	**N**	**%**
**Age**		
<40	249	14.3
41–50	465	26.8
51–60	736	42.4
>60	287	16.5
**Gender**		
Male	650	37.4
Female	1,087	62.6
**Educational level**		
Elementary-Middle school	156	9.0
High school	599	34.5
University degree	982	56.5
**Job qualification**		
Healthcare workers	1,345	77.43
Vocational	7	0.40
Technician	158	9.10
Administrative	225	12.95
Other	2	0.12
**Local Health Unit**		
Sassari	306	17.6
Olbia	260	15.0
Nuoro	202	11.6
Lanusei	75	4.3
Oristano	208	12.0
Sanluri	94	5.4
Carbonia	136	7.8
Cagliari	456	26.3
**Area** (*N* = 1,371)		
Hospital	698	50.9
Health district	673	49.1
Years of work^*^	14.2	10.7
**Years of work**		
<5	510	29.4
6–10	340	19.6
11–15	196	11.3
16–20	203	11.7
>21	488	28.1
**Employment contract**		
Fixed-term	136	7.8
Permanent	1,601	92.2
**Management role**		
No	1,434	82.6
Yes	303	17.4
**Healthcare facility managed** (*N* = 303)		
District	8	2.6
Department	6	2.0
Complex care unit	79	26.1
Simple care department unit	32	10.6
Simple unit managed by complex care unit	41	13.5
Professional engagement	116	38.3
Other	21	6.9
**Changes in job profile**		
Promotion	30	1.7
Demotion	122	7.0
No change	1,585	91.3

* *Mean and standard deviation*.

Regarding employee responses to the section on working conditions, in nine out of 13 items on work organization more than half of the respondents scored 1 or 2 on the five-point Likert scale. Items showing more dissatisfaction were “availability of training and updating tools” and “fairness and justice in the recognition and progress of career,” with 80.2% and 75.0% of respondents scoring 1 or 2 points. The distribution of responses under ‘relationships' was more heterogeneous. The highest percentage of one- or two-point scores was shown by the items “receive sufficient and non-contradictory advice from superiors” and “definition of role/responsibility,” which reached 40.3% and 39.5% respectively. Conversely, for all the mental stress items, less than half of the respondents scored one or two.

Employee suggestions were grouped into 10 areas: increase in staff availability, additional resources, greater compensation and benefits, additional employee development, additional training, effective collaboration with supervisors, better healthcare service, more flexible schedule, more rights, more equity of employee rewards. Descriptive analysis of employee suggestions (*N* = 935) shows “additional resources” to be the improvement most requested by participants (21%) followed by “effective collaboration with supervisors” (20.5%), “increase in staff availability” and “more equity of employee rewards” (both 18.6%). More details on descriptive statistics of the individual items in each category and employee suggestions are reported in Supplementary Tables 1, 2.

Regarding the section that queried employee opinion on the merger process, the vast majority of respondents scored the first item one (66.5%) or two (28.2%), expressing a poor opinion of the improvement in quality of the services offered to the population as a result of the merger, whereas participants had a better view of improvements in their own job and the department, with the majority scoring three for both items (77.7 and 67.5% respectively) (Supplementary Table 3).

### Determinants of Lower Job Satisfaction

Among socio-demographic characteristics, univariate comparisons revealed statistically significant differences according to age (*p*-value = 0.04), pre-merger LHUs (*p*-value = 0.04), working area (*p*-value < 0.001), management role (*p*-value < 0.001) and changes in job profile (*p*-value < 0.001) (Supplementary Table 4). Specifically, the highest percentages of less-satisfied employees were found among those who originally worked at the Carbonia LHU (31.6%) and those who received a demotion/downgrade (45.1%). Conversely, the lowest percentages of less-satisfied individuals were found among those employees who worked in a health district (19.5%), were older than 60 years (19.9%) and had a management role (15.2%). Employees with a poor experience in one of the six areas pertaining to working conditions more frequently reported lower job satisfaction, and the difference was statistically significant for each area (*p*-value < 0.001) (Supplementary Table 5). In particular, employees reporting higher levels of mental stress had the highest prevalence of lower job satisfaction (58.0%). Concerning employee suggestions, workers who requested additional resources showed the lowest frequency of lower job satisfaction (18.9%), whereas those who requested “more equity of employee rewards” showed the highest (47.1%), with both differences reaching statistical significance (Supplementary Table 5).

According to the results of the univariate analyses, variables initially included in the logistic regression model, which examined the associations between working conditions, socio-demographic variables and lower job satisfaction, were working conditions, sex, age, job qualifications, LHU, area (hospital or health district), years of work, management role, change in position after merger. Table 2 presents the results of the final logistic regression model after backward selection. After adjusting for potential confounders, employees more likely to experience lower job satisfaction were those with an administrative role (aOR: 2.34, 95%CI: 1.37–4.00) and a career demotion (aOR: 1.84, 95%CI: 1.11–3.03), compared to healthcare workers and employees with no change in professional profile after the merger, respectively. Working in a health district (OR: 0.72, 95%CI: 0.52–0.99) and having more years of work (OR: 0.99, 95%CI: 0.97–1.00) were associated with a decreased likelihood of lower job satisfaction. Regarding working conditions, a high degree of satisfaction in the work organization (aOR: 0.16, 95%CI: 0.08–0.34) and relationships (aOR: 0.42, 95%CI 0.25–0.70) were negatively associated with lower job satisfaction, while high levels of mental stress were positively associated with the outcome (aOR: 5.64, 95%CI: 4.16–7.64).

**Table 2:** Multivariate analysis model to identify association between factors contributing to working conditions and lower job satisfaction (*N* = 1,370).

**Variables**	**OR**	**CI 95%**	***p*** **-value**
Areas^*^			
Work organization	0.16	0.08–0.34	<0.001
Work schedule	0.93	0.45–1.90	0.837
Relationships	0.42	0.25–0.70	0.001
Environmental conditions	0.93	0.59–1.48	0.766
Physical stress	1.18	0.86–1.63	0.306
Mental stress	5.64	4.16–7.64	<0.001
**Role/job qualification**			
Healthcare workers	Ref.	–	–
Administrative employee	2.34	1.37–4.00	0.002
Vocational	12.15	0.60–249.4	0.105
Technician	0.74	0.41–1.32	0.301
Other	Omitted		
**Area**			
Hospital	Ref.	–	–
Health district	0.72	0.52–0.99	0.041
**Years of work**	0.99	0.97–1.00	0.037
**Changes in job profile**			
No change	Ref.	–	–
Demotion	1.84	1.11–3.03	0.017
Promotion	1.17	0.23–5.87	0.852

*OR, Odds Ratio; CI, Confidence Interval*.

* *For work organization, work schedule, relationships and environmental conditions, the OR refers to the more satisfied employees (75° percentile vs. other employees). For physical and mental stress, the OR refers to the less satisfied employees (25° percentile vs. other employees)*.

A second multivariate logistic regression model (Table 3) was built to investigate the association between employee requirements and lower job satisfaction. Among the 10 suggestions, the only variable found to be statistically significant was “more equity of employee rewards” with an adjusted OR of 2.30 (95%CI: 1.51–3.47). None of the socio-demographic variables showed a significant association with employee satisfaction. By contrast, a job demotion was significantly associated with lower job satisfaction (aOR: 3.40, 95%CI: 1.91–6.06).

**Table 3:** Multivariate analysis model to identify association between employee suggestions and lower job satisfaction (*N* = 741).

**Variables**	**OR**	**IC 95%**	***p*** **-value**
**Suggestions**			
Increase in staff availability	0.98	0.65–1.48	0.918
Additional resources	0.67	0.43–1.05	0.078
Greater compensation and benefits	0.86	0.50–1.49	0.549
Additional employee development	1.29	0.84–2.00	0.249
Additional training	0.90	0.54–1.48	0.671
Effective collaboration with supervisors	0.83	1.51–3.46	<0.001
Better healthcare service	0.95	0.52–1.73	0.867
More flexible schedule	1.04	0.57–1.87	0.909
More rights	0.86	0.50–1.50	0.605
More equity of employee rewards	2.30	1.51–3.47	<0.001
**Years of work**	0.99	0.98–1.01	0.413
**Sex**			
Male	Ref.	–	–
Female	1.22	0.84–1.76	0.296
**Role/job qualification**			
Administrative	Ref.	–	–
Vocational	Omitted		
Technician	0.53	0.23–1.24	0.144
Healthcare workers	0.58	0.32–1.07	0.083
Other	Omitted		
**Changes in job profile**			
No change	Ref.	–	–
Demotion	3.40	1.91–6.06	<0.001
Promotion	0.77	0.15–4.02	0.757
**Area** (*N* = 1,371)			
Hospital departments	Ref.	–	–
Territorial departments	0.61	0.42–0.88	0.009

*OR, Odds ratio; CI, Confidence interval*.

## Discussion

The aim of this study was to implement a methodology for evaluating employee satisfaction in a population of healthcare and non-healthcare workers after a merger process and to assess factors associated with lower job satisfaction, providing management with a tool for subsequent planning.

Responders showed a good level of job satisfaction overall and with regard to the merger process, with more than 75% of employees finding a moderate improvement in their job after the merger and more than 66% a moderate improvement in their ward/service. This may reflect when the survey was administered, i.e., 1 year after the merger took place, a period in which there was probably a high degree of acceptance of the necessity of organizational reconfiguration so as to improve patient services and the hospital's performance (13). However, the Hawthorne effect could have played a role in the responders because of the knowledge of survey participation. Conversely, only 5% found a moderate improvement in the quality of service offered to the population. This result is in line with the literature, which demonstrates that the first aim of a merger is cost-saving and increased efficiency rather than improvements in the quality of care (21).

The response rate was low, at 10.8% of the total employee population. Furthermore, the respondents did not accurately represent the whole staff population, according to the Sardinia LHU organizational chart. In fact, healthcare workers and administrative staff were over-represented in our sample, while technicians were under-represented. The reasons for the different response rates among different sectors of the target population are difficult to determine without further information, although the use of a web survey could be a factor. Moreover, since this is, as far as we are aware, the first survey to take into account the job satisfaction of both healthcare and non-healthcare workers, there are no other studies with which we might make a comparison (22, 23).

Administrative staff were more likely to be associated with lower job satisfaction than healthcare workers and technicians. Considering that technicians were mainly social workers, it can be hypothesized that direct involvement in patient care is associated with a greater job satisfaction (24). In our sample, employees who worked in a health districts were more satisfied than those who worked in a hospital. A possible explanation of this finding may be the difficulties experienced when working in hospital settings, such as working long shifts, dealing with unscheduled tasks or facing high levels of stress due to the emotional involvement of direct patient care (25, 26).

A statistically significant negative association was found between number of years worked and lower job satisfaction. Evidence in the literature about the effect of years worked on job satisfaction is mixed (24). However, one study has previously shown similar results in nurses, suggesting that work experience may lead to increased competence and familiarity with job tasks and thus reduce the level of occupational stress (27).

Regarding working conditions, more than half the respondents expressed a negative opinion of some aspects of work organization. The high prevalence of negative answers in this category could reflect the profound effect that the policies, procedures and contextual factors of the organization have on how staff view the quality of their work life (28). Since the merger process has a marked impact on work organization (12), it is reasonable to suppose that these results largely reflect the effects of the merger.

Among items showing more dissatisfaction, some relate to the need for recognition and career progression. These findings are consistent with the request for “more equity of employee rewards,” which is one outcome of the second multivariable model, highlighting the importance of improving the relationship between employees and superiors as a determinant of job satisfaction (29). It should also be noted that mergers can frequently cause resource reallocation with consequent changes in career pathways, possibly leading to a worse perception on equity of employee rewards (12).

In contrast to items dealing with work organization, less than half the respondents scored all mental stress items very low and mental stress was strongly associated with lower job satisfaction. It is well known that job satisfaction is linked to individual outcomes such as work-related stress and a previous study has shown that the higher the level of work-stress employees experience, the lower the job satisfaction expected (30). These findings suggest the importance of improving not only those aspects of working life with which most employees are dissatisfied, but also those more likely to be associated with job dissatisfaction.

Our study has some strengths and limitations. The main strength is the development of a valuable method for assessing the well-being of employees in a healthcare setting, which we demonstrate to be particularly useful in the context of reorganization and restructuring programs. This methodology may be a reproducible instrument for stakeholders to discover where intervention is required to improve the working conditions of healthcare workers; a healthy workplace in fact correlates with health system quality (26, 31). The limitations of our research are mostly due to the low response rate, so our sample might not be representative of the entire LHU population. Future studies should take into account the possibility of a low web-survey response rate and put in place actions to improve it. Additionally, due to the cross-sectional design of our study, causal interpretations must be made with caution. Moreover, we only analyzed a single LHU and therefore our results may have limited generalizability. However, these findings should be representative of the situation at regional level and thus could be useful for comparing job satisfaction among similar workers in the various Italian regions.

## Conclusions

To our knowledge this is the first study that assesses job satisfaction among the employee population of an LHU after a merger. Local Health Units are complex systems, as are the processes in which they are involved, and this means that any merger of LHUs is likely to have complex ramifications, including potentially detrimental effects on staff members. The exploratory analysis represented by this study is important in that it highlights challenging areas and critical working conditions affected by a merger. These areas should be further investigated over time by repeated surveys to confirm the current results and to analyse their evolution over time.

## Data Availability Statement

The raw data supporting the conclusions of this article will be made available by the authors, without undue reservation.

## Ethics Statement

The studies involving human participants were reviewed and approved by Azienda per la tutela della salute Sardegna. The patients/participants provided their written informed consent to participate in this study. Written informed consent was obtained from the individual(s) for the publication of any potentially identifiable images or data included in this article.

## Author Contributions

CI, AN, PS, AZ, MG, AS, and DM drafted the manuscript, handled the data management, and conducted the analyses. VB, GM, CM, and PV critically revised the manuscript for important intellectual content. CD, RM, GS, and FM conceived the study and critically revised the manuscript. All authors reviewed, read, and approved the final version of the manuscript.

## Conflict of Interest

The authors declare that the research was conducted in the absence of any commercial or financial relationships that could be construed as a potential conflict of interest.

## Publisher's Note

All claims expressed in this article are solely those of the authors and do not necessarily represent those of their affiliated organizations, or those of the publisher, the editors and the reviewers. Any product that may be evaluated in this article, or claim that may be made by its manufacturer, is not guaranteed or endorsed by the publisher.
