# Supplementary material for: Job Satisfaction Among Employees After a Merger: A Cross-Sectional Survey in the Local Health Unit of Sardinia Region, Italy
Source: Front Public Health. 2021 Dec 9;9:798084. doi: 10.3389/fpubh.2021.798084 (PMC8725631; doi:10.3389/fpubh.2021.798084)
Supplement: Supplementary file 1 [file Table_1.docx]

**Supplementary Table 1**. Absolute and relative frequencies of employee responses to the questionnaire on a scale from 1 to 5 (1=worst condition; 5=best condition) for the six groups of questions relating to working conditions.

|  | 1  N (%) | 2  N (%) | 3  N (%) | 4  N (%) | 5  N (%) |
| --- | --- | --- | --- | --- | --- |
|  |  |  |  |  |  |
| Work organization |  |  |  |  |  |
| Salary treatment | 216 (12.4) | 775 (44.6) | 503 (29) | 224 (13) | 19 (1.1) |
| Career opportunities | 531 (30.6) | 790 (45.5) | 293 (16.9) | 114 (6.6) | 9 (0.5) |
| Possibility to organize work | 192 (11.1) | 580 (33.4) | 616 (35.5) | 290(16.7) | 59 (3.4) |
| Ability to decide even only partially what to do | 124 (7.1) | 637 (36.7) | 657 (37.9) | 263 (15.1) | 56 (3.2) |
| Access to courses or training | 282 (16.2) | 755 (43.5) | 473 (27.2) | 188 (10.8) | 39 (2.2) |
| Opportunity to learn new things | 240 (13.8) | 767 (44.2) | 502 (28.9) | 186 (10.7) | 42 (2.4) |
| To be encouraged to have new ideas, to make suggestions for improvement | 452 (26.0) | 694 (40) | 401 (23.1) | 162 (9.3) | 28 (1.6) |
| Variability of work tasks | 264 (15.2) | 771 (44.4) | 519 (29.9) | 150 (8.6) | 33 (1.9) |
| Receive appreciation and recognition from superiors | 342 (19.7) | 471 (27.1) | 551 (31.7) | 323 (18.6) | 50 (2.9) |
| Receive unfair criticism from superiors | 68 (3.9) | 237 (13.6) | 526 (30.3) | 505 (29.1) | 401 (23.1) |
| Fairness and justice in recognition and progression of career | 598 (34.4) | 706 (40.6) | 312 (18) | 106 (6.1) | 15 (0.9) |
| Support from superiors in case of difficulties at work | 262 (15.1) | 552 (31.8) | 501 (28.8) | 333 (19.2) | 89 (5.1) |
| Availability of training and updating tools | 713 (41.1) | 679 (39.1) | 252 (14.5) | 83 (4.8) | 10 (0.6) |
| Work schedule |  |  |  |  |  |
| Flexible working hours | 656 (37.8) | 551 (31.7) | 365 (21) | 130 (7.5) | 35 (2) |
| Choice of when to take a break | 210 (12.1) | 574 (33.1) | 740 (42.6) | 178 (10.3) | 35 (2) |
| Relationships |  |  |  |  |  |
| Receive sufficient and non-contradictory advice from superiors | 184 (10.6) | 516 (29.7) | 567 (32.6) | 365 (21.0) | 105 (6.0) |
| Definition of roles/responsibilities | 187 (10.8) | 498 (28.7) | 557 (32.1) | 407 (23.4) | 88 (5.10) |
| Receive appreciation for work done from colleagues | 98 (5.6) | 302 (17.4) | 686 (39.5) | 567 (32.6) | 84 (4.8) |
| Support and help from colleagues | 55 (3.2) | 200 (11.5) | 530 (30.5) | 664 (38.2) | 288 (16.6) |
| Personal relationships with colleagues | 11 (0.6) | 90 (5.2) | 426 (24.5) | 867 (49.9) | 343 (19.8) |
| Enviromental conditions |  |  |  |  |  |
| Spaces and furnishings | 416 (24) | 476 (27.4) | 534 (30.7) | 276 (15.9) | 35 (2.0) |
| Availability of equipment | 343 (19.8) | 608 (35.0) | 572 (32.9) | 197(11.3) | 17 (1) |
| General relationships among staff | 150 (8.6) | 383 (22.1) | 636 (36.6) | 491 (28.3) | 77 (4.4) |
| Physical stress |  |  |  |  |  |
| Risk of injury or occupational disease | 114 (6.6) | 212 (12.2) | 431 (24.8) | 789 (45.4) | 191 (11) |
| Work overload | 283 (16.3) | 394 (22.7) | 705 (40.6) | 301 (17.3) | 54 (3.1) |
| Physical fatigue | 187 (10.8) | 337 (19.4) | 583 (33.6) | 539 (31.0) | 91 (5.2) |
| Uncomfortable physical position | 246 (14.2) | 327 (18.8) | 529 (30.5) | 544 (31.3) | 91 (5.2) |
| Mental stress |  |  |  |  |  |
| Risk of losing your job during the next two years | 59 (3.4) | 38 (2.2) | 67 (3.9) | 576 (33.2) | 997 (57.4) |
| Need to have special skills and/or unusual experience | 242 (14) | 567 (32.6) | 690 (39.7) | 194 (11.2) | 44 (2.5) |
| Difficulty in being able to do everything | 119 (6.9) | 276 (15.9) | 558 (32.1) | 635 (36.6) | 149 (8.6) |
| Having a lower qualification than required for the job | 133 (7.7) | 209 (12.0) | 303 (17.4) | 525 (30.2) | 567 (32.6) |
| Having higher skills and abilities than required for the job | 152 (8.8) | 328 (18.9) | 636 (36.6) | 425 (24.5) | 196 (11.3) |
| Lack of rules, too much left to own initiative | 186 (10.7) | 290 (16.7) | 415 (23.9) | 646 (37.2) | 200 (11.5) |
| In the last 30 days (20 working days), were you happy to go to work? | 255 (14.7) | 184 (10.6) | 202 (11.6) | 570 (32.8) | 526 (30.3) |
| In the last 30 days, did you or would you willingly complete a job on your own time, for example, staying after working hours? | 458 (26.4) | 292 (16.8) | 533 (30.7) | 172 (9.9) | 282 (16.2) |
| Are you happy with the results obtained at your work? | 106 (6.1) | 420 (24.2) | 722 (41.6) | 369 (21.2) | 120 (6.9) |
| Do you consider your job useful? | 19 (1.1) | 75 (4.3) | 448 (25.8) | 667 (38.4) | 528 (30.4) |
| Do you feel capable of overcoming difficulties that you may face in your job? | 10(0.6) | 61(3.5) | 733(42.2) | 689(39.7) | 244(14.1) |
| To what extent do you feel mentally tired or exhausted at the end of a normal working day? | 282 (16.2) | 478 (27.5) | 741 (42.7) | 220 (12.7) | 16 (0.9) |
| How much your work is a source of nervous tension, concern and anxiety? | 243 (14) | 350 (20.2) | 593 (34.1) | 479 (27.6) | 72 (4.1) |
| How much do you want to change the workplace, while maintaining the same type of job? | 305 (17.6) | 287 (16.5) | 306 (17.6) | 463 (26.7) | 376 (21.7) |
| How much would you like to change the type of work? | 156 (9) | 168 (9.7) | 225 (13) | 526 (30.3) | 662 (38.1) |
| Number of respondents: 1737 | | | | | |
