# Supplementary material for: Job Satisfaction Among Employees After a Merger: A Cross-Sectional Survey in the Local Health Unit of Sardinia Region, Italy
Source: Front Public Health. 2021 Dec 9;9:798084. doi: 10.3389/fpubh.2021.798084 (PMC8725631; doi:10.3389/fpubh.2021.798084)
Supplement: Supplementary file 2 [file Table_2.docx]

**Supplementary Table 2**. Employee opinions categorized by types of suggestions.

| Variables | N | % |
| --- | --- | --- |
| Increase in staff availability | 174 | 18.6 |
| Additional resources | 196 | 21 |
| Greater compensation and benefits | 106 | 11.3 |
| Additional employee development | 172 | 18.4 |
| Additional training | 132 | 14.1 |
| Effective collaboration with supervisors | 192 | 20.5 |
| Better healthcare service | 80 | 8.6 |
| More flexible schedule | 87 | 9.3 |
| More rights | 103 | 11 |
| More equity of employee rewards | 174 | 18.6 |

Number of respondents: 935
