# Supplementary material for: Job Satisfaction Among Employees After a Merger: A Cross-Sectional Survey in the Local Health Unit of Sardinia Region, Italy
Source: Front Public Health. 2021 Dec 9;9:798084. doi: 10.3389/fpubh.2021.798084 (PMC8725631; doi:10.3389/fpubh.2021.798084)
Supplement: Supplementary file 3 [file Table_3.docx]

| **Supplementary Table 3**: Absolute and relative frequencies of responses on a scale of 1 to 5 (1= worst opinion; 5= best opinion) on employer perceptions after the merger. | | | | | |
| --- | --- | --- | --- | --- | --- |
|  | 1  N (%) | 2  N (%) | 3  N (%) | 4  N (%) | 5  N (%) |
|  |  |  |  |  |  |
| After the LHU merger, was there an improvement in the services offered to the population? | 1155 (66.5) | 489 (28.2) | 79 (4.6) | 12 (0.7) | 2 (0.1) |
| After the LHU merger, was there an improvement in your job? | 28 (1.6) | 360 (20.7) | 1349 (77.7) | 0 | 0 |
| After the LHU merger, was there an improvement in amount of work of your department? | 30 (1.7) | 534 (30.7) | 1173 (67.5) | 0 | 0 |
| Number of respondents: 1737; LHU: Local Health Unit | | | | | |
|  | | | | | |
|  | | | | | |
