# Supplementary material for: Job Satisfaction Among Employees After a Merger: A Cross-Sectional Survey in the Local Health Unit of Sardinia Region, Italy
Source: Front Public Health. 2021 Dec 9;9:798084. doi: 10.3389/fpubh.2021.798084 (PMC8725631; doi:10.3389/fpubh.2021.798084)
Supplement: Supplementary file 4 [file Table_4.docx]

**Supplementary Table 4**. Univariate analysis of socio-demographic and occupational variables of respondents reporting lower job satisfaction.

| Variables | Employees with lower job satisfaction N (%) | Rest of employees N (%) | p-value |
| --- | --- | --- | --- |
| **Age** |  |  |  |
| <40 | 69 (27.7) | 180 (72.3) | 0.04 |
| 41-50 | 131 (28.2) | 334 (71.8) |  |
| 51-60 | 173 (23.5) | 563 (76.5) |  |
| >60 | 57 (19.9) | 230 (80.1) |  |
| **Gender** |  |  |  |
| Male | 146 (22.5) | 504 (77.5) | 0.09 |
| Female | 284 (26.1) | 803 (73.9) |  |
| **Educational level** |  |  |  |
| Elementary-Middle school | 33 (21.2) | 123 (78.8) | 0.40 |
| High school | 157 (26.2) | 442 (73.8) |  |
| University degree | 240 (24.4) | 742 (75.6) |  |
| **Job qualification** |  |  |  |
| Healthcare workers | 329 (24.5) | 1,016 (75.5) | 0.59 |
| Vocational | 1 (14.3) | 6 (85.7) |  |
| Technician | 36 (22.8) | 122 (77.2) |  |
| Administrative | 63 (28.0) | 162 (72.0) |  |
| Other | 1 (50.0) | 1 (50.0) |  |
| **Local Health Unit** |  |  |  |
| Sassari | 77 (25.2) | 229 (74.8) | 0.04 |
| Olbia | 74 (28.5) | 186 (71.5) |  |
| Nuoro | 40 (19.8) | 162 (80.2) |  |
| Lanusei | 11 (14.7) | 64 (85.3) |  |
| Oristano | 57 (27.4) | 151 (72.6) |  |
| Sanluri | 18 (19.2) | 76 (80.8) |  |
| Carbonia | 43 (31.6) | 93 (63.4) |  |
| Cagliari | 110 (24.1) | 346 (75.9) |  |
| **Area** (N=1371) |  |  |  |
| Hospital | 207 (29.7) | 491 (70.3) | <0.001 |
| Health district | 131 (19.5) | 542 (80.5) |  |
| **Years of work^*^** | 13.4 (0.5) | 14.4 (0.3) | 0.04 |
| **Employment contract** |  |  |  |
| Fixed-term | 30 (22.1) | 106 (77.9) | 0.45 |
| Permanent | 400 (25.0) | 1,201 (75.0) |  |
| **Management role** |  |  |  |
| No | 384 (26.8) | 1,050 (73.2) | <0.001 |
| Yes | 46 (15.2) | 257 (84.8) |  |
| **Healthcare facility managed** (N=303) |  |  |  |
| District | 0 (0.0) | 8 (100.0) | 0.49 |
| Department | 1 (16.7) | 5 (83.3) |  |
| Complex care unit | 9 (11.4) | 70 (88.6) |  |
| Simple care department unit | 3 (9.4) | 29 (90.6) |  |
| Simple unit managed by complex care unit | 6 (14.6) | 35 (85.4) |  |
| Professional engagement | 24 (20.7) | 92 (79.3) |  |
| Other | 3 (14.3) | 18 (85.7) |  |
| **Changes in job profile** |  |  |  |
| Promotion | 3 (10.0) | 27 (90.0) | <0.001 |
| Demotion | 55 (45.1) | 67 (54.9) |  |
| No change | 372 (23.5) | 1,213 (76.5) |  |
| ^*^Mean and standard deviation |  |  |  |
