# Supplementary material for: Job Satisfaction Among Employees After a Merger: A Cross-Sectional Survey in the Local Health Unit of Sardinia Region, Italy
Source: Front Public Health. 2021 Dec 9;9:798084. doi: 10.3389/fpubh.2021.798084 (PMC8725631; doi:10.3389/fpubh.2021.798084)
Supplement: Supplementary file 5 [file Table_5.docx]

| **Supplementary Table 5**. Univariate analysis of survey question areas and staff suggestions. | | | | |
| --- | --- | --- | --- | --- |
| **Areas** (N=1737) | Employees with higher (or lower) score in each area* N | Employees with lower job satisfaction N (%) | Rest of employees N (%) | p-value |
|  |  |  |  |  |
| Work organization | 428 | 12 (2.8) | 416 (97.2) | <0.001 |
| Relationships | 429 | 29 (6.8) | 400 (93.2) | <0.001 |
| Environmental conditions | 430 | 55 (12.8) | 372 (87.2) | <0.001 |
| Work schedule | 192 | 18 (9.4) | 174 (90.6) | <0.001 |
| Physical stress | 502 | 191 (38.0) | 311 (62.0) | <0.001 |
| Mental stress | 448 | 260 (58.0) | 188 (42.0) | <0.001 |
|  |  |  |  |  |
| **Suggestions** (N=935) | Employees’ suggestions N | Employees with lower job satisfaction N (%) | Rest of employees N (%) | p-value |
|  |  |  |  |  |
| Increase in staff availability | 212 | 53 (25.0) | 159 (75.0) | 0.180 |
| Additional resources | 196 | 37 (18.9) | 159 (81.1) | 0.001 |
| Greater compensation and benefits | 106 | 27 (25.5) | 79 (74.5) | 0.440 |
| Additional employee development | 172 | 59 (34.3) | 113 (65.7) | 0.070 |
| Additional training | 132 | 36 (27.3) | 96 (72.7) | 0.703 |
| Effective collaboration with supervisors | 192 | 49 (25.5) | 143 (74.5) | 0.280 |
| Better healthcare service | 80 | 20 (25.0) | 60 (75.0) | 0.449 |
| More flexible schedule | 87 | 24 (27.6) | 63 (72.4) | 0.816 |
| More rights | 103 | 33 (32.0) | 70 (68.0) | 0.422 |
| More equity of employee rewards | 174 | 82 (47.1) | 92 (52.9) | <0.001 |
| *For work organization, relationships, environmental conditions and work schedule, number of employees with higher scores (75^th^ percentile) are indicated. For physical and mental stress, number of employees with lower score (25^th^ percentile) are indicated. | | | | |
